# Software tool for 3D extraction of germinal centers

**DOI:** 10.1186/1471-2105-14-S6-S5

**Published:** 2013-04-17

**Authors:** David N Olivieri, Merly Escalona, Jose Faro

**Affiliations:** 1 School of Computer Engineering, University of Vigo, Ourense, 32004, Spain; 2 Department of Biochemistry, Genetics and Immunology, University of Vigo, Vigo, 36310, Spain; 3 Estudos Avançados de Oeiras, Instituto Gulbenkian de Ciência, Oeiras, 2781-901, Portugal

## Abstract

**Background:**

Germinal Centers (GC) are short-lived micro-anatomical structures, within lymphoid organs, where affinity maturation is initiated. Theoretical modeling of the dynamics of the GC reaction including follicular CD4^+ ^T helper and the recently described follicular regulatory CD4^+ ^T cell populations, predicts that the intensity and life span of such reactions is driven by both types of T cells, yet controlled primarily by follicular regulatory CD4^+ ^T cells. In order to calibrate GC models, it is necessary to properly analyze the kinetics of GC sizes. Presently, the estimation of spleen GC volumes relies upon confocal microscopy images from 20-30 slices spanning a depth of ~ 20 - 50 *μ*m, whose GC areas are analyzed, slice-by-slice, for subsequent 3D reconstruction and quantification. The quantity of data to be analyzed from such images taken for kinetics experiments is usually prohibitively large to extract semi-manually with existing software. As a result, the entire procedure is highly time-consuming, and inaccurate, thereby motivating the need for a new software tool that can automatically identify and calculate the 3D spot volumes from GC multidimensional images.

**Results:**

We have developed *pyBioImage*, an open source cross platform image analysis software application, written in python with C extensions that is specifically tailored to the needs of immunologic research involving 4D imaging of GCs. The software provides 1) support for importing many multi-image formats, 2) basic image processing and analysis, and 3) the *ExtractGC *module, that allows for automatic analysis and visualization of extracted GC volumes from multidimensional confocal microscopy images. We present concrete examples of different microscopy image data sets of GC that have been used in experimental and theoretical studies of mouse model GC dynamics.

**Conclusions:**

The pyBioImage software framework seeks to be a general purpose image application for immunological research based on 4D imaging. The *ExtractGC *module uses a novel clustering algorithm for automatically extracting quantitative spatial information of a large number of GCs from a collection of confocal microscopy images. In addition, the software provides 3D visualization of the GCs reconstructed from the image stacks. The application is available for public use at http://sourceforge.net/projects/pybioimage/.

## Background

During the later phase of primary immune responses to protein antigens, as well as in secondary immune responses to the same antigen, the produced antibodies display higher affinity for their antigen compared with the early phase of the response, a phenomenon known as affinity maturation [1]. The precise mechanisms responsible for this phenomenon are the subject of current intense research, and are known to take place in well-organized micro-anatomical structures, called germinal centers (GC), that develop temporarily within primary follicles of secondary lymphoid organs during immune responses to protein antigens [2].

The number of GCs and their average size increases dramatically within the first week after immunization and then start to decrease within days 10-14, so that by days 21-24 very few of them remain, while those that do have small sizes. GCs consist of a dominant population of antigen-specific B cells and smaller populations of T lymphocytes, follicular dendritic cells, and macrophages [3-5]. The antigen-specific B cells proliferate intensely, undergo somatic hypermutation in the variable region of their antibody molecules, and are subject to a poorly understood affinity-based selection process [2,6].

The long-held interest in GCs stems from being the place where a Darwinian process, involving somatic hypermutation (SHM) and selection, acts on responding B cells and their antibodies, thereby leading to memory B cell generation and to the phenomenon of affinity maturation. Because of the very high rate of SHM (10^-4 ^to 10^-3 ^per base pair and cell division), a GC reaction with an excessively long duration may not only spoil previous affinity enhancing mutations, but also generate autoreactive and even aberrant mutations leading to leukemia cells. Contrarily, because of the random character of SHM, affinity-enhancing mutations appear only several days after the activation of hypermutation, so that GC reactions with durations too short will have an ineffectual selection. As a result, it is not totally surprising that the time scale of GC reactions is regulated. The precise mechanisms that drive and control the dynamics of GCs are not presently known and is the focus of intense research.

Recently some of us [7] and others [8,9] have shown that the dynamics of GCs is controlled by follicular regulatory CD4^+ ^T (T_F_reg) lymphocytes, a newly discovered distinct subpopulation of Foxp3^+^CD4^+ ^T cells that share with follicular CD4^+ ^T helper cells the same responsiveness to the follicular chemokine CXCL13. The impact of T_F_reg on the kinetics of GC sizes was made evident in studies involving confocal microscopy analysis of murine mesenteric lymph nodes at different times after immunization [7]. Our theoretical modeling of the dynamics of the GC reaction, including T_F_reg cells, suggests that the intensity and life span of such a reaction is subject to two different controlling processes: an initial process driven by T_F_reg cells, and a later one, detectable only when the first process is too weak, controlled by follicular CD4^+ ^T helper cell maturation (JF, manuscript in preparation). In order to properly calibrate GC models with T_F_reg lymphocytes, comparisons and fits to experimentally obtained GC sizes taken at different time during the kinetics of entire process is fundamental. A sufficiently accurate study, however, requires a more exhaustive analysis with the acquisition of more time points during the immune response than previously accomplished in experiments to date. Also, such an analysis would require accurate determination of all the GC volumes obtained from these experiments.

**Figure 1:**
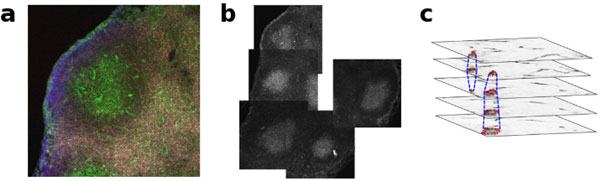
**Fluorescent confocal microscopy image of germinal centers**. (a) Confocal microscopy image showing a germinal center (green labeled cells) together with the outer zone in a draining lymph node from a mouse at day 17 after immunization. (b) The image mosaic of a larger portion of the lymph node specimen formed from individual images of germinal centers. (c) A graphical representation of the 3D stack formed from the images slices togehter with a construction of the GC volumes.

Presently, the estimation of GC volumes relies upon sectioning either the spleen or lymph nodes in several tissue samples of approximately ~ 20 - 50 *μ*m thickness and performing immunohistochemical staining. Subsequently, confocal microscopy is used to acquire images from each section at different equally spaced plane depths, usually generating more than 20-30 thin slices. These image slices are then digitized and assembled into Z-stacks, as shown in Figure 1. Finally, using a program such as ImageJ [10,11], the analysis of GC areas is performed semi-manually slice-to-slice, in the Z-stack, for subsequent 3D reconstruction. Such a tedious and highly time-consuming procedure provides a crude estimation of GC volumes. Moreover, the sheer quantity of data to be analyzed from this large set of confocal microscopy imaging is so prohibitively large, that manual or semi-manual extraction is not tenable. Indeed, for understanding the order of magnitude of data produced, a typical experiment for study GC dynamics involves the analysis of on average ~100 GCs (5-10 slices per GC) per mouse and per time point (minimum 3-4 mice per time point and 4-5 time points). As a result, there may be more than 6000 individual GC slices to be analyzed in a single experiment.

**Available software **A candidate software application for the proposed task would provide automatic measurements of all GC densities; that is, an accurate and automatic measure of the individual GC volumes, a count of the constituent cells, and subsequent visual confirmation of each GC using a three dimensional isosurface reconstruction [12]. Perhaps the two most popular representative *open-source *software tools used for post-processing of microscopy images are ImageJ [10,11] (together with a newer distribution branch, Fiji [13]) and OMERO [14-16]. Other open-source software tools for biological visualization include Vaa3D (http://www.vaa3d.org), which is a cross platform tool geared towards biological visualization of 3D/4D/5D formats, and Icy (http://icy.bioimageanalysis.org), which is another powerful image analysis software that provides a powerful environment for third party developers together with visualization software. Several commercial software applications, such as Imaris and MetaMorph, are also widely used by the biology community for performing post-processing image analysis and visualization tasks. While a complete listing or comparison of all available software solutions are beyond the scope of this paper, these applications are certainly state-of the art and highly representative of other applications with their particular advantages/disadvantages. Also, in keeping with our design philosophy, we have focused more upon open-source analysis tools for comparing our software and algorithms.

In the case of OMERO, this is a large client/server application, designed to provide centralized access of images from a disk server, and provides many types of analysis as well as data annotation and workflow. While OMERO has a large user base, and many analysis extensions, it presently lacks the ability to automatically perform segmentation of objects such as GCs in 3D (also referred to as 3D spot volumes) and does not provide a 3D output that allows for visual checking of the accuracy of the borders of the detected GCs. Moreover, there is no provision in their roadmap for the addition of these difficult, yet important features [14].

Fiji/ImageJ, is a multi- platform Java-based application written for the desktop that uses the powerful ImageJ image analysis library for a microscopy specific application. It features extendible plug-in module support, scripting in multiple languages, and supports a large collection of image formats used the microscopy community. In an independently developed branch, Fiji, provides many new powerful analysis extensions; an example of which is image registration techniques [17-19], for stitching multidimensional images from low-level autocorrelation of features. Nonetheless, it also does not contain the capability for automatic segmentation of volumes with constituent cells, as envisioned in this work.

In summary, with respect to segmenting and extracting GC volumes, the microscopy software applications and algorithms that we have evaluated either (a) lack sufficient information about the segmented dimensions, (b) underestimate the number of objects segmented due to the difficulty of selecting the appropriate input parameters, (c) provide only gross estimates of areas/volumes, or (d) simply do not provide the desired functionality for automatically obtaining GC volumes. As such, with respect to extracting GC volumes, no single software tool exists, to our knowledge, able to perform the proposed automated tasks and that meets all requirements desired.

While ImageJ and Fiji have a large user base and provide the ability to write customized plug-ins in various programming languages, we decided from the onset to deviate from this standard development course in order to develop our own microscopy infrastructure, written in python and called **pyBioImage**. While motivated by several reasons, the principle advantage of this design choice is to leverage the growing software base for scientific computing with powerful and efficient numerical and visual libraries recently made available in the python community. Given the power of the python C-extension API, available libraries, and the ability for rapid and robust open software development, other microscopy software application have recently emerged, albeit with slightly different scientific goals, but based upon a similar python/C design philosophy. Two recent open source tools also written in python and C/C++, which have recently been reported in the literature for microscopy applications, are IOCBioMicroscope [20] (focused upon deconvolution of microscopy images) and BioImageXD [21].

### Implementation

Our software suite, **pyBioImage**, is a cross-platform bio-imaging application, written in Python and makes use of low level C code exposed through the Python C-extension API. The application supports multiple data formats and provides visualization and analysis of standard multi-dimensional image data. For the work described in this paper, we have developed a set of algorithms implemented either in pure python or as python/C-extension modules, that form a core feature called *ExtractGC*, which is specifically tailored for automatically extracting GC volume statistics and visualization from a collection of 3D confocal fluorescent microscopy image stacks. These images are highly magnified regions of tissue samples taken from secondary lymphoid organs. The set of such images from tissue specimens may be used to reconstruct a 3D mosaic, consisting of several GCs, and thereby making it possible visualize a large section of the organ in question. Our analysis software module *ExtractGC*, which is part of the more general **pyBioImage **application, uses a pseudo-recursive segmentation algorithm for performing simultaneous pixel level clustering in all directions *xyz *of a complete image stack. Our segmentation technique is based upon a general segmentation algorithm, often referred to as *spot *finding algorithm in the context of fluorescent microscopy, first described and implemented by Goldberg *and col*. [22].

**Figure 2:**
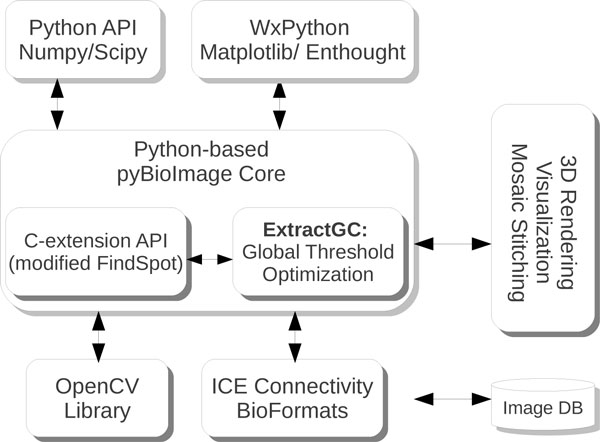
**The architecture**. The figure shows the relation between the ExtractGC module and other software components that makes up the architecture of pyBioImage. The core application of the pyBioImage application is written in Python with the graphical user library wxPython. The ExtractGC module consists of python classes, used for high level processing, and low-level pixel operations performed in C, exposed through the Python C-extension API. Numerical algorithms, image analysis, image I/O, the graphical user interface and data visualization leverage the use of powerful open-source libraries.

In order to maintain the goal of cross-platform interoperability, the selection and design of the libraries used, as well as our software suite **pyBioImage **(and in particular *ExtractGC*) depend upon several standard open source software libraries, as shown in Figure 2. In particular, in order to perform low-level pixel operations as efficiently as possible, we have written several python C-extension modules. We use Numpy and Scipy for several post-processing numerical routines. For image analysis operations, we use the OpenCV library. The graphical interface is based upon wxPython, and 2D visualization with specialized bindings with Matplotlib and the Enthought Traits library (code.enthought.com/). For 3D reconstruction, we have written python bindings for the well known powercrust algorithm [23], and visualization rendering is presently performed with with the GeomView (http://www.geomview.org), however we are presently incorporating the use of VTK (http://www.vtk.org).

Many file formats for confocal microscopy are based upon variants of Tiff, or at least the ability to include multiple images with the same file. For standard Tiff files, we have used a python based wrapper of the standard libtiff library. In order to connect with LOCI BioFormats [24], that is provided through a Java jar library module, we use the Internet Communication Engine (ICE) (http://www.zeroc.com) which provides a drop-in C/C++ connectivity for I/O module.

### Workflow and interface

**Figure 3:**
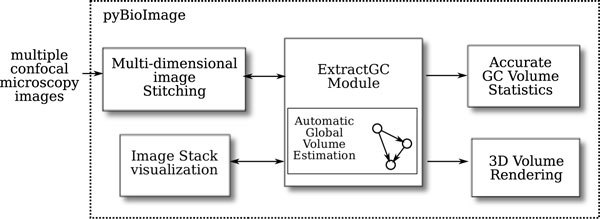
**The workflow**. ExtractGC is based upon an easy to use, yet productive workflow for quickly obtaining GC size and volume statistics at the lab bench. Given a set of confocal microscopy images, pyBioImage provides the option of direct visualization, image mosaic construction, or direct analysis with the ExtractGC module. An automatic optimization algorithm iteratively selects the best input parameters for extracting GC volumes. From the output of the analysis, the user may interact with the data via a 3D visualization.

Figure 3 illustrates the workflow of our software suite **pyBioImage **with *ExtractGC *for the specific task of obtaining detailed statistics and visualizations of GC volumes. In particular, a set of acquired confocal microscopy images are read/loaded into the application according to the appropriate format with efficient memory management. At this point, either the individual image stacks may be analyzed for obtaining GC volumes, or a mosaic may be obtained for subsequent analysis.

For extracting GC volumes, an initial seed value for the threshold and minimum spot size are provided by the user. The optimal global threshold is found with a simulated annealing optimization algorithm, by using this initial seed together with other parameters. This will produce the optimal 3D bounding surface together with statistics for all GCs that pass the maximum size specified by the input parameter. As with general stochastic global optimizers, further flexibility towards optimal solutions can be explored by adjusting a subset of input parameters. Full 3D renderisation of all or selected GC volumes may be obtained interactively by the user.

### Segmentation algorithm for extracting germinal center volumes

Broadly speaking, segmentation algorithms decompose an image into distinct parts for recognizing objects of interest. These algorithms can be divided into three groups: statistical feature-based, region-growing, and boundary methods [25,26]. For multidimensional images, feature based and boundary methods use image registration algorithms [27] to associate image pixels of one image to those of another. There are many techniques for accomplishing this task, including pixel-wise comparisons, cross-correlations, and scale invariant feature-based methods. These techniques have been extensively studied and applied to multi-dimensional medical and microscopy imaging for reconstructing volumes from different z-stack slices. Region growing methods perform segmentation by low-level pixel assembly, subject to some condition related to the pixels intensities of nearby neighbors. For multidimensional microscopy images, the FindSpot algorithm described by I. Goldberg [22], has been shown to be effective for constructing spot volumes, which are the bright/dark regions of interest, by recursively obtaining correspondence between neighboring pixels on the same and different image slices. By manually providing threshold and geometric constraints, the algorithm can efficiently encounter 3D continuous object volumes within and throughout the multidimensional image. Given the power of this method, our GC volume extraction software uses the core part of this algorithm together with several practical software modifications as well as additional algorithm details, described below.

### Optimal global threshold

Two fundamental parameters of the findspot algorithm (as developed by Goldberg *and col*.) are the pixel threshold *t_h_*, which determines which pixels are allowed into a contiguous cluster, and the minimum cluster size *s*_min _(or spot size), which provides a final cut-off on contiguous volume region. The threshold may be a global parameter or based upon the mean pixel (or even more sophisticated statistical-based methods, which for our purpose are not effective). With fluorescent microscopy, the intensity is directly proportional to the amount of B-cell membrane marker or receptor molecules, which is relatively homogeneous throughout the volume. Thus, it is sensible that a global threshold should be used since it will provide the most accurate indication of the amount of cells of a particular type at a particular z-slice. Also, a proper segmentation of the GC areas on each slice will be sensitive to an optimal selection of the initial values of *t_h _*and *s*_min_, where each depends upon the other.

**Figure 4:**
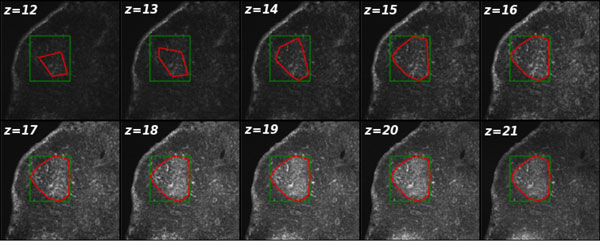
**GC borders obtained with the ExtractGC algorithm**. Different z-stack slices of the GC from Figure 1a are shown together with the performance of the segmentation algorithm. The green box represents the maximum bounding box, while the red curve is the convex hull enclosing all interior pixels above the desired intensity threshold for forming continuous clusters in 3D.

First, it is useful to understand the effect of the global threshold *t_h _*and *s*_min _parameters upon the final segmentation of GC volumes, and in particular why this selection is non-trivial. For segmenting the central part of a GC, as seen in Figure 4, slice 19, a particular threshold will perform well; however for the image slices at the extremes (in the z-plane), it becomes unclear which parameter values lead to the best segmentation results. Indeed, if the threshold is too low, pixel clusters will be unnecessarily too large (possibly selecting the entire image). However, if the threshold is too high, or optimized to the center of the GC where the fluorescent contrast is max, then on higher/lower z-stack slices, the GC borders, and hence GC volumes, will be underestimated. With respect to the selection of the minimum spot size parameter, a small minimal spot size will result in many pixel clusters that are not germinal centers.

For the specific case of segmenting GCs from multidimensional images, we can use biological information to guide the choice of an appropriate objective function. In particular, it is well known that by staining tissue samples with flourochrome-tagged peanut agglutinin, GCs will be brightly labeled throughout the volume and consist of fluorescently marked B cells that are involved in the immune response, while adjacent regions are characterized by a pronounced dark ring or halo. This dark outer ring zone is due to both follicular B cells not participating in the immune response (and, therefore, are not antibody-flourochrome labeled) and to T and dendritic cells of the adjacent T-cell zone, which are also unlabeled.

**Figure 5:**
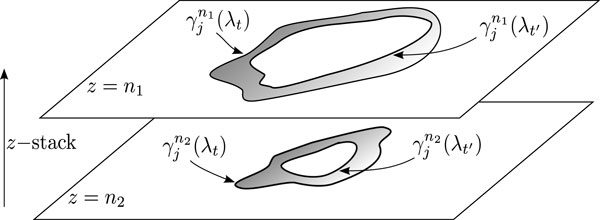
**Model parameters**. Graphical representation of two segmented regions representing the regions of GC shown on two different slices *n*_1 _and *n*_2 _used for defining the objective function for automatically optimizing the input parameter λ.

**Figure 6:**
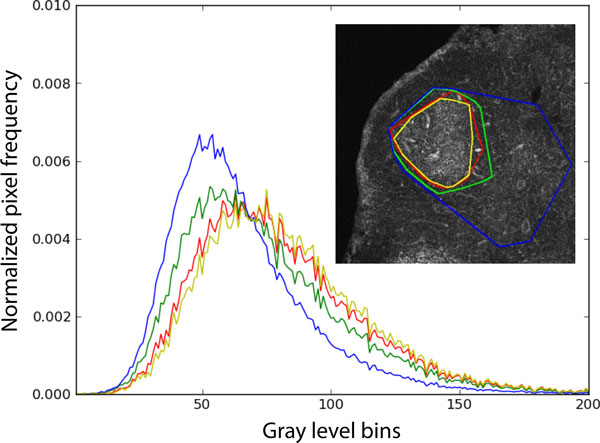
**Histogram comparison of segmented regions**. The normalized histograms for four different segmented regions $\gamma_{j}^{n}$, for a particular GC and slice *n *using four different input parameter values $\lambda_{t}$ are shown. Inset, *j*-th GC and slice *n *displaying the corresponding different segmentations. The objective function L  tries to minimize the difference between histograms, while at the same time maximize the areas on different image slices.

Given this nearly universal observed GC structure, an ideal segmentation algorithm for GCs will include all pixels up to and including the border adjacent to the dark halo zone. Since the global threshold parameter of our algorithm directly controls this segmentation, our algorithm optimizes this choice of threshold that segments the GC by the use of an iterative procedure, driven by a simulated annealing algorithm that minimize the objective function, applied to all z-slices. This objective function seeks a minimum in the sum of pixel-tone histogram differences for all z-slices, between different values of the input parameters, while at the same time strongly penalizing solutions that give rise to segmented borders outside the GC region. The algorithm for optimizing the input parameters can be formalized by referring to Figure 5 as follows. First, let $\theta_{i}\left(i=1\cdots m\right)$ represent the set of *m *input parameters to be optimized. We denote the set of values of these parameters at the iteration step *t *as $\lambda_{t}=\left\{\theta_{i}^{t}\right\}$. Next, we consider GC segmentation regions obtained from the findspot algorithm. The segmentation region of the *j*-th GC on the *n*-th image slice and at iteration step *t *is denoted by $\gamma_{j}^{n}\left(\lambda_{t}\right)$. Similarly, the segmentation region of the *j*-th GC region on the *n*-th slice at iteration *t*' is given by $\gamma_{j}^{n}\left(\lambda_{t^{\prime }}\right)$. From this, we can obtain the fraction of the number of points above the threshold *N_f _*in the annular region between $\gamma_{j}^{n}\left(\lambda_{t}\right)$ and $\gamma_{j}^{n}\left(\lambda_{t^{\prime }}\right)$ as compared to the total number of pixels *N_t _*in that annular region, as *N_f _*/*N_t_*. Moreover, for each of these regions, we can obtain the pixel-tone histogram, denoted $H\left(\gamma_{j}^{n}\left(\lambda_{t}\right)\right)\equiv H_{j}^{n}\left(t\right)$. The histograms for different segmented regions$\gamma_{j}^{n}$, for a given GC and a particular slice *n *corresponding to different input parameters $\lambda_{t}$ are shown in Figure 6. As can be seen from this figure, the difference between histograms decreases as the segmented regions are closer. An optimal solution, found from the optimal input parameters $\lambda_{t}^{*}$ would produce a segmentation that wraps tightly around the GC. Conversely, the value of the input parameters $\lambda_{t}$ should tend to maximize the individual *areas *of the segmented regions on all z-stack slices of each GC. Thus, the objective function should penalize those values of the input *λ_t _*that eliminate areas at the extremes of the GC volumes where the pixel intensities are at the limit of threshold. This tradeoff provides a convex objective function for applying an optimization strategy.

First, we use the Bhattacharyya histogram distance metric

$$D\left(H_{t},H_{t^{\prime }}\right)=\sqrt{1-\frac{\sum_{k,k^{\prime }}\sqrt{H_{t}\left(k\right)\cdot H_{t^{\prime }}\left(k^{\prime }\right)}}{\sqrt{\sum_{k}H_{t}\left(k\right)\cdot \sum_{k^{\prime }}H_{t^{\prime }}\left(k^{\prime }\right)}}}$$

where *H_t _*and *H*_*t*'_, represent $H_{j}^{n}\left(\lambda_{t}\right)$ and $H_{j}^{n}\left(\lambda_{t^{\prime }}\right)$, and *k *and *k*' represent the individual bins in each histogram, respectively.

Now, let $a_{j}^{n}\left(\lambda_{t}\right)$ be the area of the segmentation region $\gamma_{j}^{n}\left(\lambda_{t}\right)$, that is, corresponding to the *j*-th GC at slice *n *using input parameters *λ *at iteration *t*, and let

$$A_{j}^{n}\left(\lambda_{t},\lambda_{t^{\prime }}\right)=\frac{\left|a_{j}^{n}\left(\lambda_{t}\right)+a_{j}^{n}\left(\lambda_{t^{\prime }}\right)|-|a_{j}^{n}\left(\lambda_{t}\right)-a_{j}^{n}\left(\lambda_{t^{\prime }}\right)\right|}{\left|a_{j}^{n}\left(\lambda_{t}\right)+a_{j}^{n}\left(\lambda_{t^{\prime }}\right)\right|}$$

which is a symmetric function, since$A_{j}^{n}\left(\lambda_{t},\lambda_{t^{\prime }}\right)=A_{j}^{n}\left(\lambda_{t^{\prime }},\lambda_{t}\right)$. Then, we can define the objective function $L_{j}$ for the *j*-th GC as follows:

$$L_{j}\left(\lambda_{t},\lambda_{t^{\prime }}\right)=\frac{\sum_{n}\text{exp}\left[\alpha \;D\left(H_{j}^{n}\left(\lambda_{t}\right),H_{j}^{n}\left(\lambda_{t^{\prime }}\right)\right)\right]}{\varepsilon +\beta \sum_{n}A_{j}^{n}\left(\lambda_{t},\lambda_{t^{\prime }}\right)}$$

where *∈ *is a small nonzero constant that we insert to prevent division by zero error, while *α *and *β *are arbitrary constants (we have used *α ~ *0.001 and *β *= 1.0) that could be useful for controlling the strength of either the histogram difference or the sum over areas, respectively. With this function, the optimization is then with respect to the input parameters $\lambda_{t}=\left\{\theta_{i}^{t}\right\}$, that is, $\partial L_{j}/\partial \theta_{i}=0$. Notice that if the areas $a_{j}^{n}\left(\lambda_{t}\right)$and $a_{j}^{n}\left(\lambda_{t^{\prime }}\right)$on each slice *n *for each segmentation region *j *are very different —either because $a_{j}^{n}\left(\lambda_{t}\right)$ is much larger than $a_{j}^{n}\left(\lambda_{t^{\prime }}\right)$ (or vice-versa) or because, suddenly, aj(λt)=0(or aj(λt′)=0) due to the disappearance of the contour at slice *n*—, then $A_{j}^{n}\left(\lambda_{t},\lambda_{t^{\prime }}\right)\to 0$and $L_{j}\left(\lambda_{t},\lambda_{t^{\prime }}\right)$ grows to very large values, thereby penalizing $L_{j}$. Conversely, if $a_{j}^{n}\left(\lambda_{t}\right)=a_{j}^{n}\left(\lambda_{t^{\prime }}\right)$ (or are very similar) then $A_{j}^{n}\left(\lambda_{t},\lambda_{t^{\prime }}\right)\to 1$.

**Figure 7:**
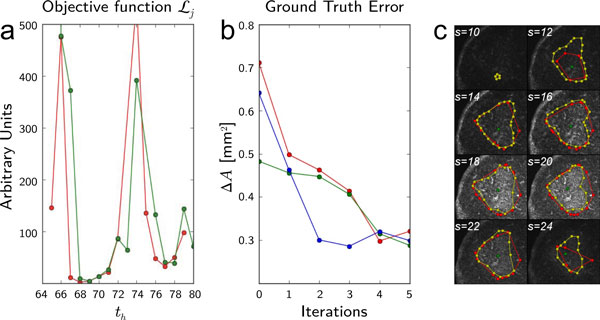
**Ground truth comparisons**. (a) Two hyperplane cuts of the multidimensional objective function $L_{j}\left(\lambda_{t},\lambda_{t^{\prime }}\right)$, with respect to *λ*= (*t_h_, s*_min_), with *s*_min _held constant and two values of the arguments, $t_{h}^{\prime }=t_{h}+1$ (red curve) and $t_{h}^{\prime }=t_{h}+2$ (green curve); values of the constants in $L_{j}$ were *α *= 0.001 and *β *= 1.0. (b) Various iterations of the optimization algorithm, showing the values of the area difference between ground truth and the calculated borders. The area difference is obtained by summing the difference of areas for all image slices. The plot shows that despite the initial *λ*, the algorithm converges towards a constant non-zero area difference. (c) Visual inspection of borders obtained with an optimal solution λ* compared with the ground truth, superposed on the corresponding germinal center image.

In general, the function $L_{j}\left(\lambda_{t},\lambda_{t^{\prime }}\right)$ is a nonlinear multidimensional function with many local minimima, many of which are not ideal solutions. In order to understand the behavior of $L_{j}$ as a function of $\lambda_{t}$ and $\lambda_{t^{\prime }}$, Figure 7(a) shows two hyperplane cuts with *s*_min _for different values of the threshold *t_h_*. For constructing this plot, we chose consecutive values of the threshold, with $t_{h}^{\prime }=t_{h}+1$ (red curve), and $t_{h}^{\prime }=t_{h}+2$ (green curve). As can be seen, in both planes, the function experiences a dramatic global minimum for the optimal solution.

From this objective function, we use a simulated annealing algorithm that efficiently samples the space of all possible $\lambda_{t}$ in order to find the optimal set of input parameters,$\lambda^{*}$, given by:

$$\lambda^{*}=\underset{\lambda_{t}}{\text{argmin}}\;L_{j}\left(\lambda_{t},\lambda_{t^{\prime }}\right)$$

In order to show how robuts our optimization algorithm is with respect to the choice of initial input parameters, Figure 7(b) shows the difference in accumulated area (which is related to the GC volume) between the calculated and ground truth value for several iterations of the algorithm for three separate initial values of *λ*. In these studies, the *ground truth *determination was obtained from manual inspection by an expert. Figure Figure 7(c) shows a comparison, superposed on a particular Germinal Center image, between borders obtained with optimal parameter solution, λ*, using our algorithm and the ground truth border obtained by manual determination.

Since the original *findspot *algorithm finds all contiguous clusters of pixels throughout a volume, connected regions can be filled with holes. By using a convex hull algorithm, or more sophisticated computational geometry algorithms based upon alpha shapes, we can represent and visualize the 3-dimensional GC volumes with the outer bounding surface. Nearby artifacts due to outliers points may be present, distorting the volume estimate, and should be corrected. We eliminate outliers by a simple heuristic algorithm that determines the full distance matrix between all points on the contour and determines whether the distance between each point and all others is greater than 2 × *σ *value of all other inter point distances (where *σ *is the standard deviation). Conversely, we can find the geometric center and determine whether a point is 2 × *σ *from that center.

**Optimal stitching **Our software **pyBioImage **also contains a module for automatic stitching of multi-dimensional images, similar to that found in ImageJ. Side-by-side z-stack images of draining lymph nodes were acquired to allow 3D reconstructions of larger organ areas. Due to the large amount of image stacks, we developed our own software algorithms that used information from the microscope position and accelerated the task of forming large image mosaics, referred to as image stitching, from adjacent z-stacks acquisitions.

For matching adjacent image stacks, our algorithm uses a fast implementation of the Fourier phase correlation technique for achieving image registration at the borders of adjacent (and overlapping) images. For blending adjacent images, we use a nonlinear pyramid scheme together with pixel intensity scaling for matching potential differences in acquisition exposures. The implementation of our algorithm is available in our cross-platform **pyBioImage **package, available at the public repository (sourceforge.net/projects/pybioimage/). Information about the installation, documentation, and other software modules (whose description is beyond the scope of this paper), can also be found in the package distribution.

#### 3D reconstruction

**Figure 8:**
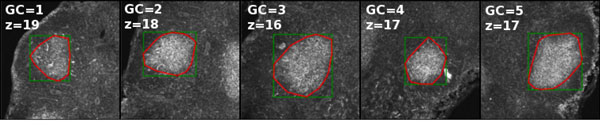
**Examples of borders extracted for different germinal centers**. *ExtractGC *analysis on five images of different germinal centers taken from the same specimen shown in the mosaic of Figure 1b. The images show the border (red curves) at slices in the center of each germinal center.

Another capability of the *ExtractGC *module is the ability to accurately visualize the GC volumes in 3D. The reconstruction of the set of borders pixels obtained from each z-stack slice is used for constructing an isosurface with a computational geometry algorithm, called Powercrust, described by Amenta, Choi and Kolluri [28,29]. We have provided a full set of python bindings to the original open-source C-language implementation of these authors in order to easily expose the core algorithm to our application, **pyBioImage**. The output of powercrust, with the points obtained from the findSpot algorithm in the prior phase, is a set of files that specify the polygons and their vertex locations in 3D that define the maximum bounding surface. Presently, we have maintained the original powercrust related .off extension file structure which can be visualized with Geomview, an interactive 3D viewing program for Unix. An example 3D renderization of a GC is shown in Figure 8.

Details of the 3D rendering algorithm are as follows: the algorithm takes point samples from a 3D object's surface and produces both a surface mesh and an approximate medial axis. The powercrust algorithm is based on the Medial Axis Transformation (MAT) that provides a complete description of the object's shape through maximally inscribed discs. Together with the Voronoi diagram computation its duality, and its weighted adaptations, the powercrust algorithm produces the so-called power diagram. Then, the algorithm can be described briefly with the following steps [30]: (1) given a collection of sampled points, a bounding box is used to enclose what shall be the 3D object, (2) a Voronoi Diagram is computed and pole computation for each sample point is made, (3) each pole is analyzed and labeled with its relation to the Voronoi surfaces, and finally (4) the algorithm provides output of the powercrust and powershape parameters, that can be used for constructing polygons of the external surface.

While there are several other fundamental algorithms for 3D reconstruction, including Alpha Shapes (a generalization of the convex hull algorithm by Edelsbrunner), Marching Cubes, Voronoi-based algorithms, and Delaunay-based algorithms, we found the Crust/Powercrust algorithms the most effective for our application.

## Results and discussion

The architecture of pyBioImage, together with the *ExtractGC *module, is designed to provide a productive and intuitive workflow for the experimental and theoretical biologist for extracting accurate GC statistics.

### Germinal center image acquisition

In order to test our software, we applied our algorithms to a set of GC image data acquired with typical experimental conditions. In particular, Balb/c mice maintained in SPF facilities were immunized intraperitoneally with 20 *μ*g of OVA (Sigma, St Louis, USA) previously run through a DetoxyGel column (Pierce, Rockford, USA) in 2.0 mg of endotoxin-free aluminum hydroxide (alum, Alu-gel-S, Serva, Heidelberg, Germany). Seventeen days after immunization the draining lymph nodes were excised and fixed with PFA (Sigma). 50 *μ*m vibratome sections from fixed tissue were stained with the following primary antibodies: rabbit anti-CD3 (Abcam), rat anti-IgM-TxRd (SouthernBiotech, Birmingham, USA), and PNA-FITC (Vector, Burlingam, USA). Anti-rabbit immunoglobulin-alexa647 (Invitrogen, Carlsbad, USA) was used as secondary antibody.

Once the regions of interest were located, 35 images were acquired at 1.43*μ*m z-steps, using a LSM710 confocal microscope (Zeiss, Jena, Germany) equipped with a 20 × (0,80 NA, Zeiss) objective. Several images were acquired across a relatively large section of the specimen, such that each image contained at least one GC, and the set of all images formed a mosaic (with an irregularly ordered tiling).

### Evaluation

From the data, prepared as described above, we analyzed four independent data sets that represent magnified regions of small sections of lymph nodes. For each specimen, 5 GCs were imaged independently, with a slight overlap of the nearby image, so that a mosaic could be formed. The images consisted of 4-color channels, were 512 × 512, and contained an average of 30 z-stack slices. We used our algorithm to automatically collect GC statistics by loading all the images in the directory and providing initial input parameter guesses for the pixel intensity threshold and the minimum spot size: *λ *= (*t_h_, s*_min_). Results of extracting GCs for different datasets are shown in Figure 4, showing the contour encountered of the GC region at different z-stack slices.

The algorithms described are efficient, requiring no special hardware, and can run on any modern computer system. In order to appreciate the typical running times, we ran the algorithm on a standard laboratory computer (Intel Pentium D CPU 2.80GHz, with 2G Memory), and execution times to process multi-dimensional images with sizes 512 × 512 × 35 never exceeded from 1.2 s and the execution time for the optimization step was always below 0.5 s for different image sizes.

**Figure 9:**
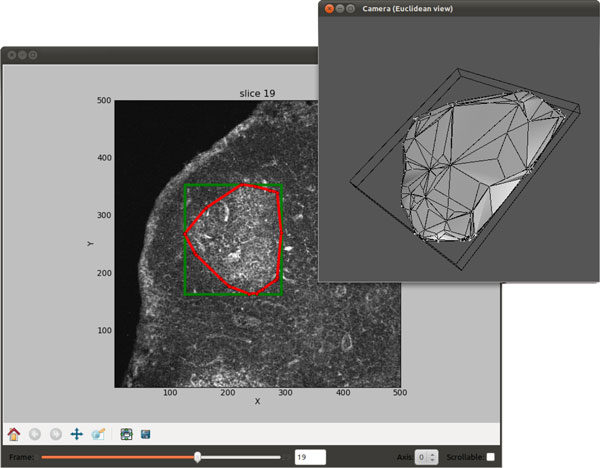
**Screenshots of the application with the 2D/3D visualization of germinal centers**. The 2D visualization allows the user to maneuver through the slices by using a slider. The 3D visualization provides interactive manipulation, as well as a wide array of rendering options.

Figure 9 illustrates the visualization features of pyBioImage/ExtractGC. The 2D window, allows the user to maneuver through the image stacks slice by slice, with the segmented contour superposed on the image. A 3D visualization allows the user to interactively manipulate the GC volume from all angles, and provides a more accurate calculation of the GC volume.

## Conclusions

Our application, **pyBioImage **with the ExtractGC module provides fully automatic and accurate estimates of GC volumes from an arbitrarily large collection of multidimensional images. The framework pyBioImage leverages the relatively recent availability of high quality scientific software based upon python for rapid development of complex image and computation. As such, our application is positioned to tackle several problems described in this paper not provided by standard open-source solutions, such as Fiji/ImageJ. The *ExtractGC *module is a relevant bioinformatics tool that should be of interest to scientists working with confocal and 2-photon microscopy imaging and has also served to be a proof of concept module for integrating specific applications within our general software framework. Given the usefulness of the *ExtractGC *module, we are presently planning to also release a version of the algorithm for both the ImageJ as well as OMERO projects.

## Availability and requirements

Project name: e.g. **pyBioImage **package

Project home page: http://sourceforge.net/projects/pybioimage/

Operating system(s): Platform independent

Programming language: phyton, C

Other requirements:

License: GNU GPL

Any restrictions to use by non-academics: license needed

## List of abbreviations

T_F_reg: follicular regulatory CD4^+ ^T lymphocytes; GC: germinal center; 2D: 2-dimensional; 3D: 3-dimensional; 4D: 4-dimensional.

## Competing interests

The authors declare that they have no competing interests.

## Authors' contributions

JF and DO conceived, designed and developed the study, DO and ME implemented the software code, JF, DO and ME wrote the manuscript. All authors read and approved the final manuscript.

## Declarations

This article has been published as part of *BMC Bioinformatics *Volume 14 Supplement 6, 2013: Selected articles from the 10th International Conference on Artificial Immune Systems (ICARIS). The full contents of the supplement are available online at http://www.biomedcentral.com/bmcbioinformatics/supplements/14/S6.
